# Association of upper limb motor function with muscle tone changes and quality of life in the subacute phase after stroke: a prospective cohort study

**DOI:** 10.3389/fneur.2026.1857094

**Published:** 2026-05-25

**Authors:** Denis Moskiewicz, Małgorzata Kołodziej, Michał Mikulski, Iwona Sarzyńska-Długosz

**Affiliations:** 1 Department of Physiotherapy in Motor Organ Dysfunction and Kinesiology, Faculty of Physiotherapy, Wroclaw University of Health and Sport Sciences, Wroclaw, Poland; 2 Rehabilitation Department, T. Marciniak Lower Silesian Specialist Hospital – Emergency Medicine Centre, Wroclaw, Poland; 3 Department of Physiology and Biomechanics, Faculty of Physical Education and Sport, Wroclaw University of Health and Sport Sciences, Wroclaw, Poland; 4 EGZOTech Sp. z o.o., Gliwice, Poland; 5 Department of Neurology and Neurodegenerative Diseases, Institute of Psychiatry and Neurology, Warsaw, Poland

**Keywords:** muscle tone, myotonometry, neurorehabilitation, quality of life, stroke rehabilitation, subacute stroke, upper limb motor function

## Abstract

**Background:**

Changes in muscle tone after stroke may influence upper limb (UL) motor recovery and quality of life (QoL), but their relationship remains unclear. This study evaluated associations between muscle tone changes, UL motor function, and QoL in patients undergoing subacute stroke rehabilitation.

**Methods:**

This prospective cohort study is a secondary analysis of data from a randomized controlled trial including 58 patients in the subacute phase after stroke. UL motor function was assessed using the Fugl-Meyer Assessment (FMA-UE) and Box and Blocks Test (BBT), muscle tone using myotonometry, and QoL using the EuroQol-5 Dimensions-5 Levels (EQ-5D-5L) questionnaire. Correlations between changes in these parameters were analysed.

**Results:**

Most changes in muscle tone were not significantly associated with improvements in upper limb motor function or quality of life. Only isolated, weak correlations were observed, including negative associations between changes in the total score of the Fugl–Meyer Assessment for Upper Extremity and changes in relaxation time of the flexor digitorum superficialis muscle measured using myotonometry (*r* = −0.32, *p* = 0.016) and the logarithmic decrement (elasticity) of the wrist flexor muscles (flexor carpi radialis and flexor carpi ulnaris) measured using myotonometry (*r* = −0.26, *p* = 0.046), as well as between changes in the BBT and changes in the creep parameter of the latissimus dorsi muscle measured using myotonometry (*r* = −0.31, *p* = 0.017).

**Conclusion:**

UL motor recovery may occur largely independently of changes in muscle tone and is more closely associated with QoL. Rehabilitation should prioritise motor control and functional performance rather than focusing primarily on tone reduction.

## Introduction

Globally, the consequences of stroke remain one of the most serious challenges for healthcare systems, as it continues to be one of the leading causes of death and long-term disability in the adult population (1, 2). Each year, approximately 12 million people worldwide experience a stroke, of whom over 7 million die, and a substantial proportion of those who survive the acute phase experience lasting consequences leading to disability, particularly in low- and middle-income countries (3).

Impairment of motor function, affecting approximately 77% of patients after stroke (4), is the most common clinical manifestation, significantly limiting daily functioning and requiring multidisciplinary rehabilitation aimed at restoring motor performance (5). Among the causes of motor deficits, paresis (6), defined as reduced muscle strength, is most frequently identified (6). Alongside spasticity, it constitutes one of the main symptoms of corticospinal tract damage. In the case of stroke, it often takes the form of hemiparesis of varying severity, affecting one side of the body (7).

Hemiparesis occurs in approximately 65% of patients after stroke (8). Paresis is observed both in the UL, where it occurs in over 70% of patients (9) (with muscle strength averaging 59% of normative values), and in the lower limb (LL), with strength reduced to approximately 69% of normative values (10). It is estimated that 30 to 66% of patients with UL paresis experience functional impairment persisting for at least 6 months after the event (11). Early and specialised rehabilitation plays a crucial role in improving motor performance, and patients with better initial UL function have up to approximately 15 times greater chances of achieving full recovery compared to those with more severe deficits (12).

Another important factor limiting the recovery of motor function after stroke is spasticity, which often coexists with paresis and represents a significant therapeutic challenge (13). In the initial period after stroke, reduced muscle tone (hypotonia) is frequently observed as a consequence of damage to the central nervous system (CNS), particularly the pyramidal tracts and subcortical structures such as the lentiform nucleus (14). Damage to these areas leads to disturbances in the regulation of muscle tone, manifested by decreased activity of alpha motor neurons and inhibition of muscle reflexes, contributing to persistent hypotonia in the early post-stroke phase (15). As the condition progresses, due to an imbalance between inhibitory and excitatory processes at the level of the spinal cord and higher neural centres, disinhibition of the stretch reflex occurs, leading to the development of increased muscle tone (hypertonia), the most common form of which is spasticity (16, 17). The pathomechanisms underlying this phenomenon include, among others, damage to the corticospinal tract resulting in impaired inhibitory control over alpha motor neurons, as well as neuroplastic changes occurring in the spinal cord (16, 18).

Spasticity is estimated to occur in 4–42.6% of patients after stroke (19). Among patients with paresis after a first stroke, the prevalence of spasticity is 39.5%, with approximately 9.4% developing a severe form that leads to significant functional limitations and difficulties in rehabilitation (20). Spasticity has a substantial negative impact on patient functioning and the course of rehabilitation, resulting in reduced motor abilities (21, 22), decreased quality of life (23), and lower levels of independence among stroke survivors (24). Increased muscle tone also leads to higher costs of hospitalisation, care, and medication (25).

One of the key elements in the diagnosis, planning, and monitoring of rehabilitation progress in patients with spasticity is its assessment. In clinical practice, the most commonly used scale is the Modified Ashworth Scale (MAS), which is used to evaluate resistance of muscles to passive stretching (26). Despite its widespread use, the MAS is characterised by limited sensitivity in detecting changes in muscle tone and low inter-rater reliability (27, 28). An alternative tool with higher validity is the Tardieu Scale, which, unlike the MAS, takes into account the effect of passive movement velocity on muscle tone assessment, allowing for a more effective differentiation between the dynamic component of spasticity and contracture (29, 30). In the original version of the scale, the angle of the first resistance (R1), maximum range of motion (R2), and the quality of muscle reaction (X) were assessed; however, the lack of standardised stretching velocity limited its reliability (31). The modified version (MTS) introduced standardised measurement protocols, precise definitions of stretching velocities, and angle measurement standards, thereby increasing the reliability and clinical value of the tool (32). Despite these methodological improvements, both MAS and MTS remain subjective tools, which limits their objectivity and reproducibility (33).

In recent years, increasing attention has been paid to objective, instrument-based methods for assessing muscle tone (34). MyotonPRO is an advanced, non-invasive device for the precise measurement of biomechanical muscle properties, such as tone, stiffness, and elasticity, characterised by high reliability of results (35). In the context of stroke rehabilitation, measurements obtained using MyotonPRO enable more accurate monitoring of biomechanical muscle changes, as well as evaluation of the effectiveness of therapeutic interventions, which may contribute to optimisation of the rehabilitation process (36).

Previous studies indicate that the correlations between muscle tone and motor function after stroke are complex and multifaceted (37). Changes in muscle tone during the first year after stroke significantly affect UL mobility, leading to deterioration in function, pain, and reduced range of motion (38). Findings regarding the correlation between changes in muscle tone and improvement in UL function in patients after stroke remain inconclusive (39, 40). There is a need to further investigate the correlation between actual improvement in motor function, changes in muscle tone, and overall quality of life in individuals after stroke (41).

Considering the vital role of muscle tone in the rehabilitation process and its impact on motor function and quality of life in patients after stroke, it is essential to thoroughly examine the correlations between these factors using both subjective and objective assessment methods. The aim of the present study was to evaluate the association between improvement in UL motor function and changes in muscle tone in patients in the subacute phase after stroke, as well as to analyse their correlation with quality of life.

## Materials and methods

### Material—population (P)

This study constitutes a prospective cohort study with a secondary analytical approach based on data derived from a previously conducted randomized controlled trial. The analysis included 58 patients hospitalised in the Rehabilitation Department of the T. Marciniak Lower Silesian Specialist Hospital – Emergency Medicine Centre in Wroclaw, who met the inclusion and exclusion criteria. Participants were randomly assigned to two equal therapeutic groups: an experimental group (robot-assisted UL rehabilitation – Luna EMG) and a control group (conventional physiotherapy conducted by a therapist). No statistically significant differences were found between the groups in terms of rehabilitation outcomes (42). Therefore, in the present study, the entire group was analysed as a homogeneous cohort in order to assess the correlation between improvement in UL motor function, changes in muscle tone, and quality of life in patients in the subacute phase after stroke.

The study group included 34 males (59%) and 24 females (41%), with a mean age of 64.3 ± 9.8 years. Detailed demographic and clinical characteristics of the study participants are presented in Table 1.

**Table 1:** Demographic and clinical characteristics of the study participants.

Variable	Entire group (*n* = 58)	Ischemic stroke (*n* = 39)	Haemorrhagic stroke (*n* = 19)
Age (years), M ± SD	64.3 ± 9.8	63.8 ± 10.2	65.4 ± 9.1
Sex, *n* (%) – female	24 (41%)	15 (38%)	9 (47%)
Sex, *n* (%) – male	34 (59%)	24 (62%)	10 (53%)
Affected side, *n* (%) – right	30 (52%)	19 (49%)	11 (58%)
Time since stroke (days), median (IQR)	35 (28–44)	34 (28–42)	37 (30–45)
FMA-UE score T1, M ± SD	27.6 ± 9.2	26.9 ± 8.8	29.0 ± 10.1
MAS score T1, M ± SD	2.1 ± 0.7	2.0 ± 0.6	2.3 ± 0.8
BBT score T1, M ± SD	14.8 ± 6.1	14.5 ± 5.9	15.4 ± 6.6

FMA-UE – fugl-meyer assessment for upper extremity; MAS – modified ashworth scale; BBT – box and blocks test; M – mean; SD – standard deviation; IQR – interquartile range; *n* – sample size.

### Inclusion criteria

The inclusion criteria were as follows: individuals of both sexes, aged 18 to 90 years, within up to 6 weeks after a first ischemic or haemorrhagic stroke documented by hospital discharge records, for whom this was the only cerebrovascular event. A stable medical condition allowing participation in active rehabilitation was required, as well as a moderate, moderately severe, or severe motor deficit of the directly affected UL [classification based on the Fugl-Meyer Assessment for Upper Extremity (FMA-UE) ≤ 94 (43)], absence of severe cognitive impairment [a total score of 23 points or higher on the Mini-Mental State Examination (44)], and written informed consent for participation in the study and therapy.

### Exclusion criteria

The exclusion criteria were as follows: fixed, permanent contractures within the UL; marked spasticity of UL muscles defined as a Modified Ashworth Scale (MAS) score ≥3 in at least one muscle group (45); significant visual and auditory impairments; severe swallowing and respiratory disorders; and a confirmed diagnosis of myasthenia gravis or myasthenic syndrome.

### Therapeutic protocol

The therapeutic protocol was implemented by physiotherapists and occupational therapists in the Rehabilitation Department of the T. Marciniak Lower Silesian Specialist Hospital – Emergency Medicine Centre in Wroclaw. The total duration of the implemented therapy was 2 h per day, 5 days per week, for 6 weeks.

Diagnostic procedures were conducted on the day of initiation of the therapeutic protocol, after 3 weeks from the start of the protocol, after 6 weeks from the start of the protocol, and 3 weeks after completion of the therapeutic protocol.

There were no expectations regarding the magnitude of improvement. The study was conducted after obtaining approval from the Senate Committee for Research Ethics at the University School of Physical Education in Wroclaw (No. 4/2023).

The PICO framework (46) was used to formulate the clinical questions and design the study.

### Intervention (I)

The intervention consisted of UL training performed either with robotic assistance or therapist assistance, depending on group allocation, and was based on intensive, repetitive practice in accordance with the recommendations of Lang et al. (47).

**Figure 1:**
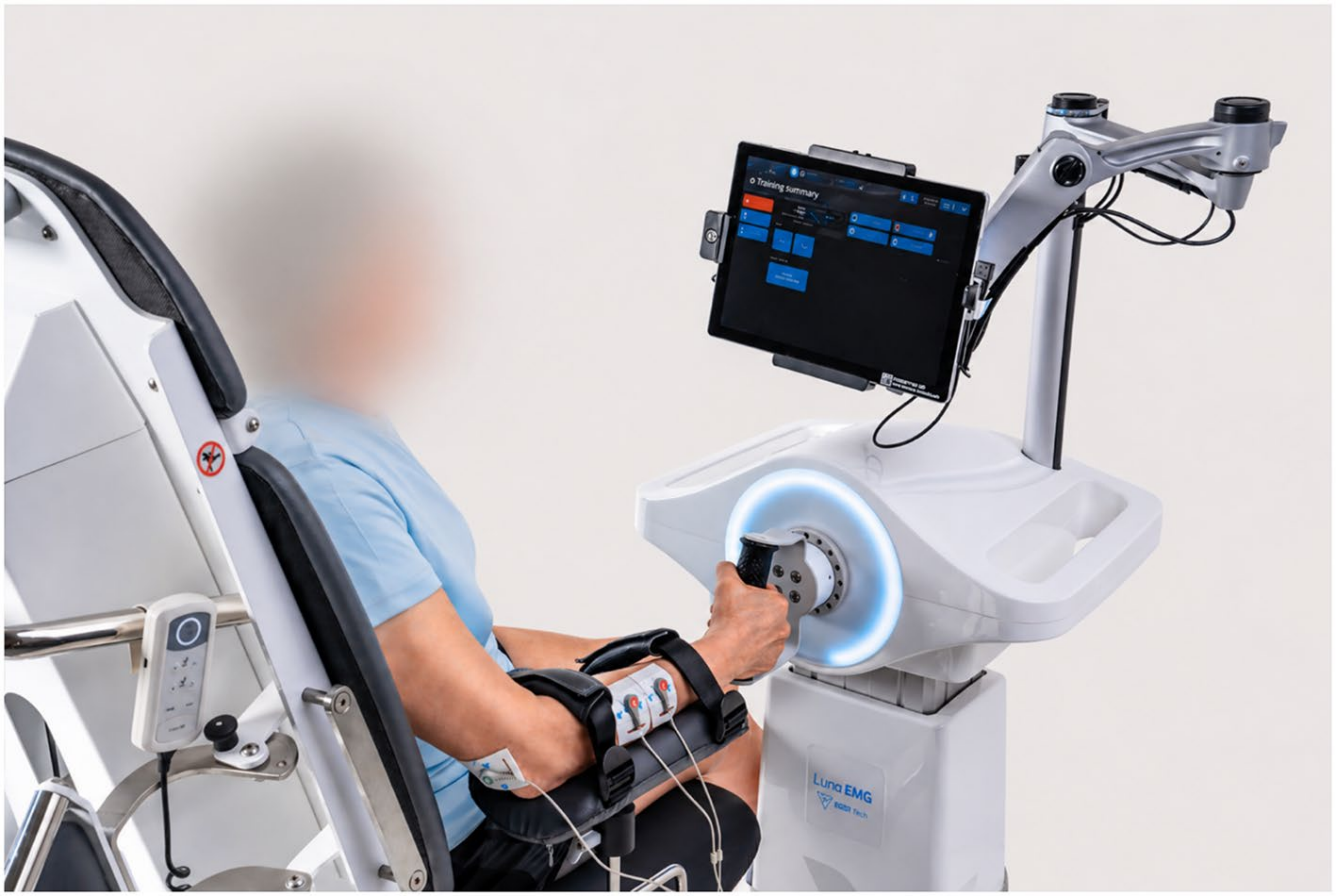
The LUNA EMG system used for upper limb rehabilitation. Surface EMG electrodes detect muscle activation and trigger assisted movement, providing real-time visual biofeedback.

In the experimental group, training was supported by the LUNA EMG system (EGZOTech, Poland), a device designed for UL rehabilitation that combines EMG-based biofeedback with interactive, task-oriented training, an approach shown to support motor recovery after stroke (Figure 1).

The system detects muscle activation using surface electromyography (EMG) and enables assisted movement once a predefined activation threshold is reached (48, 49). Surface electrodes were placed over selected UL muscles (*anterior deltoid*, *infraspinatus*, *triceps brachii*, and *brachioradialis*) in accordance with SENIAM guidelines (50). When EMG activity exceeded an individually set threshold (approximately 30% of maximal voluntary contraction), the device initiated a predefined movement trajectory and provided real-time visual biofeedback to facilitate motor learning.

Robotic training included task-specific exercises targeting proximal UL movements (shoulder flexion, external rotation, elbow extension, and forearm supination), performed in repetitive sets of approximately 50 repetitions per movement.

In the control group, participants performed comparable exercises with therapist assistance, matching the type of movement, number of repetitions, and overall training structure. The intervention protocol ensured a comparable training dose between groups, with the only difference being the mode of assistance (robot-assisted vs. therapist-assisted).

### Comparison (C)

The results were analysed jointly, without division into intervention groups. Although participants were initially randomized into two intervention groups, the primary objective of the present study was not to compare treatment effects but to investigate correlations between changes in muscle tone, motor function, and quality of life across the entire cohort.

The original randomized design ensured methodological rigor and balanced baseline characteristics; however, since no significant differences in rehabilitation outcomes were observed between groups in the primary analysis, pooling the data was considered appropriate for the purposes of the current correlational analysis. This approach increased statistical power and allowed for a more comprehensive evaluation of relationships between the studied variables.

### Outcome measures (O)

The following research tools were used for the quantitative assessment of the study objectives:

### Assessment of motor performance of the paretic UL

UL motor function was evaluated using the Fugl-Meyer Assessment for Upper Extremity (FMA-UE) (51, 52) and the Box and Blocks Test (BBT) (53).

### Assessment of muscle tone of the paretic UL

The MyotonPRO device was used to assess the mechanical properties of muscles. All measurements were performed with the muscles in a passive and relaxed state. The probe was applied perpendicularly to the skin over the muscle belly, with a constant pre-compression force (~0.18 N), followed by a brief mechanical impulse (0.4 N) inducing natural damped oscillations of the muscle tissue. At each measurement point, a series of 5 impulses was applied, and the mean value was used for analysis. The following parameters were recorded: oscillation frequency (F) corresponds to muscle tone, stiffness (S) to resistance to deformation, logarithmic decrement (D) to elasticity, relaxation time (R) to the ability of muscle to recover its shape, and creep (C) to viscoelastic behaviour (54, 55).

Assessments were conducted for the following muscle groups of the affected UL: shoulder girdle elevators (mainly *trapezius pars descendens* and *levator scapulae*), shoulder adductors, internal rotators, and extensors (mainly *latissimus dorsi*), shoulder external rotators (mainly *infraspinatus*), shoulder abductors (mainly *deltoid muscle*), elbow flexors in forearm supination (mainly *biceps brachii*), elbow flexors in a neutral forearm position (mainly *brachioradialis*), elbow extensors (mainly *triceps brachii*), forearm pronators (mainly *pronator teres*), wrist flexors (*flexor carpi radialis* and *flexor carpi ulnaris*), and finger flexors (mainly *flexor digitorum superficialis* and *flexor digitorum profundus*).

In this study, the prefix “Myo-” refers to muscle parameters measured using the MyotonPRO device, followed by the abbreviation of the assessed muscle: Bic – *biceps brachii*, Tric – *triceps brachii*, Brarad – *brachioradialis*, Delt – *deltoid muscle*, Lat/Do – *latissimus dorsi*, Infra – i*nfraspinatus*, Up.Tra – *upper trapezius*, FDS – *flexor digitorum superficialis*, Pro-Te – *pronator teres*, F.Ca – wrist flexors (*flexor carpi radialis* and *flexor carpi ulnaris*), and the specific biomechanical parameter.

Measurements were performed at standardized anatomical landmarks over the muscle belly, with participants in a relaxed supine or sitting position, depending on the assessed muscle. The grouping of muscles was based on typical patterns of post-stroke spasticity in the UL.

The MyotonPRO device is a reliable and valid tool for assessing biomechanical muscle properties in both healthy individuals and neurological populations, including patients after stroke. A recent systematic review reported high intra-rater reliability of myotonometry in stroke survivors, with mean intraclass correlation coefficients (ICC) of approximately 0.91 for upper limb muscles and 0.79 for lower limb muscles (56).

In post-stroke populations, myotonometry serves as an objective complement to commonly used clinical scales such as the Modified Ashworth Scale (MAS) and Modified Tardieu Scale (MTS), which are subjective and demonstrate limited responsiveness (41, 57).

However, myotonometry reflects passive biomechanical muscle properties and does not directly assess the neural components of spasticity (58). Accordingly, in the present study, myotonometry was used to objectively quantify muscle tone-related parameters during rehabilitation.

### Assessment of quality of life

QoL was evaluated using the EQ-5D-5L questionnaire (59), which enables assessment across five key domains: mobility, self-care, usual activities, pain/discomfort, and anxiety/depression. Each domain is rated on a five-level scale, where level 1 indicates no problems and level 5 indicates extreme difficulty or inability to perform the activity.

### Relationship to prior publication

The present study represents a secondary analysis of data derived from a previously conducted randomized controlled trial. This article constitutes an independent analysis of the same clinical cohort that was used in a previously published study comparing the effectiveness of robot-assisted rehabilitation and conventional therapy, in which no significant differences between groups in terms of rehabilitation outcomes were demonstrated.

The aim of the present study was to analyse the entire cohort collectively, focusing on the correlation between changes in UL motor function (FMA-UE, Box and Blocks Test) and changes in UL muscle tone parameters measured objectively using the MyotonPRO device, as well as quality of life assessed with the EQ-5D-5L questionnaire.

The analyses included correlations between changes in the studied parameters between the first and the final measurement, as well as associations between baseline muscle tone values and the extent of changes in motor function and quality of life during rehabilitation.

The presented results complement the findings of the conducted interventional study by providing an analysis of biomechanical, functional, and quality-of-life correlations during rehabilitation in patients in the subacute phase after stroke.

### Statistical analysis

Data distribution was verified using the Shapiro–Wilk test. Since normal distribution was not confirmed for most variables at both measurement points, the median and interquartile range (IQR) were used for statistical description of the data, and differences between paired samples were tested using the Wilcoxon signed-rank test. Effect size (ES) for differences was interpreted as small if 0.1 ≤ ES < 0.3, medium if 0.3 ≤ ES < 0.5, and large if ES ≥ 0.5 (60). For differences that showed normal distribution, mean changes between measurements along with 95% confidence intervals were calculated. Correlations between changes were assessed using Pearson’s r coefficient, as the distributions of change scores (*Δ*) approximated normality, whereas Spearman’s rank correlation coefficient was applied for analyses involving baseline variables, with effect size interpreted according to Cohen’s thresholds, where 0.1 ≤ *r* < 0.3 indicates a small effect, 0.3 ≤ *r* < 0.5 a medium effect, and r ≥ 0.5 a large effect (61).

Correlations between baseline muscle tone and the extent of changes in motor function and quality of life, due to the lack of normality of baseline data, were assessed using Spearman’s rank correlation coefficient (*ρ*). All statistical analyses were performed using TIBCO Statistica® 13.3.0 (StatSoft Poland). The results were accepted to be statistically significant at *p* < 0.05.

## Results

Significant changes were observed in UL motor function and quality of life. For most muscle tone parameters, no differences between measurements were confirmed, with the exception of several variables indicated in Table 2. Due to the extensive dataset, which included, among others, 50 variables characterising muscle tone measured at two time points, only those variables for which significant differences were identified at *p* < 0.05 are presented in Table 2. Statistics for the full dataset are provided in Appendix 1, Table A2.

**Table 2:** Changes in muscle tone parameters, UL motor function, and quality-of-life assessment in patients after stroke undergoing rehabilitation.

	Pre-test		Post-test					
	Mdn	IQR	Mdn	IQR	*p*	ES	ΔM ± SD	95%CI
Myo-Bic-D	1.59	0.28	1.46	0.46	0.049	0.26	−0.12 ± 0.41	(−0.23; −0.01)
Myo-Bic-R	18.90	6.40	22.10	4.70	0.003	0.39	2.26 ± 5.59	(0.79; 3.73)
Myo-Tric-D	1.19	0.38	1.61	0.66	<0.001	0.58	0.32 ± 0.45	(0.2; 0.44)
Myo-Brarad-F	18.20	2.10	18.50	2.10	0.027	0.29	0.31 ± 1.23	(−0.01; 0.64)
Myo-Lat/Do-D	1.19	0.43	1.54	0.74	<0.001	0.58	0.38 ± 0.53	(0.23; 0.52)
Myo-Lat/Do-R	19.00	6.40	20.70	5.10	0.035	0.28	1.54 ± 5.46	(0.11; 2.98)
Myo-Lat/Do-C	1.71	0.46	1.78	0.51	0.027	0.29	0.15 ± 0.46	(0.03; 0.27)
Myo-Infra-F	18.40	2.00	17.90	2.00	0.039	0.27	−0.39 ± 1.31	(−0.73; −0.04)
Myo-Infra-D	1.32	0.71	1.57	0.50	<0.001	0.48	0.31 ± 0.58	(0.16; 0.46)
Myo-FDS-D	1.20	0.39	1.50	0.46	0.001	0.44	0.22 ± 0.46	(0.1; 0.34)
Myo-FDS-R	18.05	3.60	20.35	6.10	<0.001	0.47	2.61 ± 5.45	(1.17; 4.04)
Myo-F.Ca-D	1.55	0.34	1.29	0.34	<0.001	0.52	−0.26 ± 0.43	(−0.37; −0.15)
Myo-F.Ca-C	1.36	0.57	1.73	0.55	0.001	0.42	0.23 ± 0.5	(0.1; 0.36)
FMA-UE-Ttl	27.00	18.00	42.50	12.00	<0.001	0.87	13.72 ± 4.53	(12.53; 14.91)
Box n Blocks	3.00	12.00	16.00	12.00	<0.001	0.85	9.71 ± 6.14	(8.09; 11.32)
EQ-5L-Por	3.00	1.00	2.00	2.00	<0.001	0.82	−1.24 ± 0.68	(−1.42; −1.06)
EQ-5L-Sam	3.00	1.00	2.00	2.00	<0.001	0.82	−1.33 ± 0.71	(−1.51; −1.14)
EQ-5L-Z/C	4.00	1.00	3.00	1.00	<0.001	0.83	−1.21 ± 0.61	(−1.37; −1.05)
EQ-5L-B/D	2.00	1.00	2.00	2.00	0.002	0.40	−0.4 ± 0.79	(−0.61; −0.19)
EQ-5L-N/P	3.00	1.00	2.00	1.00	<0.001	0.78	−1.07 ± 0.67	(−1.25; −0.89)
EQ-5L-%Zd	40.00	20.00	73.50	20.00	<0.001	0.87	33.41 ± 8.01	(31.31; 35.52)
EQ-5L-S.I	0.50	0.27	0.41	0.25	<0.001	0.83	−0.11 ± 0.08	(−0.13; −0.09)

Mdn – median, IQR – interquartile range, *p* – *p*-value – result of the Wilcoxon test, ES – effect size, ΔM±SD – mean change and standard deviation of change, 95% CI – confidence interval, FMA-UE-Ttl – total score of upper limb motor function in the Fugl–Meyer Assessment Upper Extremity, Box n Blocks – Box and Block Test (gross manual dexterity test), EQ-5L – EuroQol 5 Dimensions 5 Levels questionnaire: EQ-5L-Por – mobility, EQ-5L-Sam – self-care, EQ-5L-Z/C – usual activities, EQ-5L-B/D – pain/discomfort, EQ-5L-N/P – anxiety/depression, EQ-5L-%Zd – self-rated health (EQ-VAS), EQ-5L-S.I – EQ-5D-5L summary index. Myo – biomechanical muscle parameters measured using the MyotonPRO device: F – frequency, S – stiffness, D – logarithmic decrement (elasticity), R – relaxation time, C – creep. Muscle abbreviations: Bic – biceps brachii, Tric – triceps brachii, Brarad – brachioradialis, Delt – deltoid, Lat/Do – latissimus dorsi, Infra – infraspinatus, Up.Tra – upper trapezius, FDS – flexor digitorum superficialis, Pro-Te – pronator teres, F.Ca – wrist flexors (flexor carpi muscles).

All analysed correlations, including both statistically significant and non-significant results, are presented in detail in Tables and Supplementary materials. The text highlights the most relevant findings for clarity of presentation.

Mean changes in motor function (FMA-UE-Ttl and Box n Blocks) and in most muscle tone parameters, with the exception of Myo-Bic-D, Myo-Infra-F, and Myo-F.Ca-D, indicated an increase in the values of these variables. For all quality-of-life variables, the mean change was negative, indicating improvement in quality of life across the analysed domains, while the self-rated health index (EQ-5L-%Zd) nearly doubled (Table 2).

**Figure 2:**
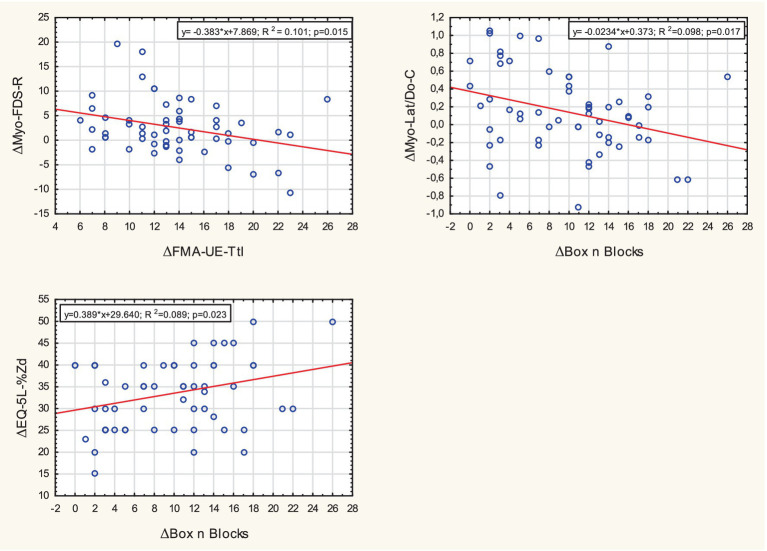
Correlations between changes in selected muscle tone parameters, upper limb motor function, and quality of life in post-stroke patients undergoing rehabilitation. ΔFMA-UE-Ttl – change in upper limb motor function assessed with the Fugl–Meyer Assessment for the Upper Extremity; ΔMyo-FDS-R – change in relaxation time of the flexor digitorum superficialis muscle measured using myotonometry; ΔBox and Blocks – change in manual dexterity assessed with the Box and Blocks Test; ΔEQ-5D-5L-%Zd – change in self-rated health (EQ-VAS) from the EuroQol-5D-5L questionnaire; *R*^2^ – coefficient of determination.

Changes in most muscle tone parameters were not correlated with changes in UL motor function or quality of life. Only a few associations were identified, characterised by small or moderate effect sizes (Table 3). Selected correlations between changes in muscle mechanical properties and functional outcomes are presented in Figure 2. Only selected correlations are presented graphically for clarity, while all analysed correlations are reported in detail in Table 3 and Supplementary materials.

**Table 3:** Correlations (Pearson’s *r*) for mean changes between the first and last measurement of muscle tone parameters, UL motor function, and quality-of-life assessment in patients after stroke undergoing rehabilitation.

	ΔFMA-UE-Ttl	ΔBox n Blocks	ΔEQ-5L-Por	ΔEQ-5L-Sam	ΔEQ-5L-Z/C	ΔEQ-5L-B/D	ΔEQ-5L-N/P	ΔEQ-5L-%Zd	ΔEQ-5L-S.I
ΔMyo-Bic-D	−0.02	−0.15	−0.14	−0.04	0.13	−0.17	−0.04	0.04	−0.18
ΔMyo-Bic-R	−0.04	−0.08	0.03	0.20	0.12	−0.04	0.00	0.19	−0.07
ΔMyo-Tric-D	−0.08	−0.05	0.01	−0.10	−0.05	0.13	0.18	−0.05	0.13
ΔMyo-Brarad-F	0.07	−0.04	0.15	0.02	−0.02	−0.12	0.11	−0.05	0.12
ΔMyo-Lat/Do-D	−0.04	−0.14	−0.09	−0.08	−0.11	−0.03	0.13	−0.02	0.02
ΔMyo-Lat/Do-R	0.05	0.01	−0.02	**−0.29**	−0.08	0.12	0.12	0.17	−0.22
ΔMyo-Lat/Do-C	−0.02	**−0.31**	−0.15	−0.24	0.04	−0.13	0.11	0.12	**−0.35**
ΔMyo-Infra-F	−0.03	−0.16	0.07	−0.09	**0.26**	−0.15	−0.05	−0.05	−0.02
ΔMyo-Infra-D	0.15	0.12	0.06	0.14	−0.05	−0.12	0.01	0.03	−0.04
ΔMyo-FDS-D	−0.05	0.07	0.08	−0.02	−0.08	**−0.32**	−0.15	0.01	−0.23
ΔMyo-FDS-R	**−0.32**	**−0.28**	0.02	0.11	0.25	0.06	−0.01	−0.16	0.15
ΔMyo-F.Ca-D	**−0.26**	−0.06	0.13	0.13	0.16	−0.17	−0.12	−0.05	0.08
ΔMyo-F.Ca-C	0.00	−0.23	−0.01	0.05	0.11	−0.20	−0.04	−0.01	−0.13
ΔEQ-5L-Por	−0.11	**0.32**	1.00	0.12	−0.20	−0.07	0.04	−0.13	**0.31**
ΔEQ-5L-Sam	−0.02	−0.11	0.12	1.00	**0.32**	−0.25	−0.16	−0.22	0.25
ΔEQ-5L-Z/C	−0.05	**−0.28**	−0.20	**0.32**	1.00	0.01	−0.08	**−0.33**	0.03
ΔEQ-5L-B/D	−0.11	−0.02	−0.07	−0.25	0.01	1.00	0.25	−0.13	0.23
ΔEQ-5L-N/P	−0.01	−0.13	0.04	−0.16	−0.08	0.25	1.00	−0.25	0.08
ΔEQ-5L-%Zd	0.14	**0.29**	−0.13	−0.22	**−0.33**	−0.13	−0.25	1.00	−0.14
ΔEQ-5L-S. I	**−0.33**	0.11	**0.31**	0.25	0.03	0.23	0.08	−0.14	1.00

Bold values indicate statistically significant correlations at *p* < 0.05. Δ – mean change between the first and the last measurement; *r* – Pearson correlation coefficient. FMA-UE-Ttl – total score of upper limb motor function in the Fugl–Meyer Assessment Upper Extremity; Box n Blocks – Box and Block Test (gross manual dexterity test). EQ-5L – EuroQol 5 Dimensions 5 Levels questionnaire: EQ-5L-Por – mobility; EQ-5L-Sam – self-care; EQ-5L-Z/C – usual activities; EQ-5L-B/D – pain/discomfort; EQ-5L-N/P – anxiety/depression; EQ-5L-%Zd – self-rated health (EQ-VAS); EQ-5L-S.I – EQ-5D-5L summary index. Myo – biomechanical muscle parameters measured using the MyotonPRO device: F – frequency; S – stiffness; D – logarithmic decrement (elasticity); R – relaxation time; C – creep. Muscle abbreviations: Bic – biceps brachii; Tric – triceps brachii; Brarad – brachioradialis; Delt – deltoid; Lat/Do – latissimus dorsi; Infra – infraspinatus; Up.Tra – upper trapezius; FDS – flexor digitorum superficialis; Pro-Te – pronator teres; F.Ca – wrist flexors (flexor carpi muscle).

Changes in FMA-UE-Ttl were negatively associated with changes in Myo-FDS-R (*r* = −0.32, *p* = 0.016) and Myo-F.Ca-D (*r* = −0.26, *p* = 0.046) and were not correlated with changes in any other muscle tone parameters. Similarly, changes in Box n Blocks were weakly and negatively correlated only with changes in two muscle tone parameters: Myo-Lat/Do-C (*r* = −0.31, *p* = 0.017) and Myo-FDS-R (*r* = −0.28, *p* = 0.033). This indicates that greater changes in the indicated muscle tone parameters may be associated with smaller improvements in UL motor function.

Changes in quality of life across individual domains were correlated to a small extent with changes in only three muscle tone parameters: in the self-care domain with ΔMyo-Lat/Do-R (*r* = −0.29, *p* = 0.028), in the usual activities domain with ΔMyo-Infra-F (*r* = 0.26, *p* = 0.047), and in the pain/discomfort domain with ΔMyo-FDS-D (*r* = −0.32, *p* = 0.014). Smaller changes in the level of problems with self-care and pain/discomfort were weakly associated with larger ΔMyo-Lat/Do-R and ΔMyo-FDS-D, respectively, while smaller changes in problems with usual activities were associated with smaller ΔMyo-Infra-F. Changes in the EQ-5L-S.I index were negatively correlated with ΔMyo-Lat/Do-C (*r* = −0.35, *p* = 0.007). With respect to UL motor function, greater changes in ΔFMA-UE-Ttl were associated with smaller changes in the EQ-5L-S.I quality-of-life index (*r* = −0.33, *p* = 0.013), whereas greater changes in Box n Blocks were correlated with greater changes in mobility (*r* = 0.32, *p* = 0.017) and self-rated health (*r* = 0.29, *p* = 0.026), as well as with smaller changes in problems with usual activities (*r* = −0.28, *p* = 0.035).

Possible associations between changes in UL motor function and quality of life with all baseline muscle tone parameters were examined, regardless of the significance of their changes in subsequent measurements (Table 4). Changes in UL motor function (ΔFMA-UE-Ttl) showed positive and moderate correlations with baseline values of Myo-Tric-1S, Myo-Tric-1D, Myo-Delt-1C, and Myo-F.Ca-1D, and a negative correlation with Myo-Up.Tra-1D. In contrast, changes in ΔBox n Blocks were negatively correlated with all muscle tone parameters labelled with F and S, with strong associations observed for Myo-Up.Tra-1S and Myo-F.Ca-1S (|*ϱ*| ≥ 0.50, *p* < 0.001).

**Table 4:** Correlations (Spearman’s *ϱ*) between baseline muscle tone parameters and changes in UL motor function and quality-of-life assessment in patients after stroke undergoing rehabilitation.

Initial variable:	ΔFMA-UE-Ttl	ΔBox n Blocks	ΔEQ-5L-Por	ΔEQ-5L-Sam	ΔEQ-5L-Z/C	ΔEQ- 5L-B/D	ΔEQ-5L-N/P	ΔEQ-5L-%Zd	ΔEQ-5L-S.I
Myo-Bic-1F	0.10	**−0.32**	−0.05	0.11	0.00	0.03	−0.11	−0.15	0.09
Myo-Bic-1S	0.16	**−0.41**	−0.13	0.18	0.16	−0.08	−0.12	−0.12	0.04
Myo-Bic-1D	0.03	0.05	0.12	0.01	−0.09	0.15	−0.06	0.05	0.15
Myo-Bic-1R	0.03	0.06	−0.07	−0.17	−0.12	0.08	0.08	−0.17	0.03
Myo-Bic-1C	−0.08	−0.07	0.05	0.06	0.07	0.05	−0.11	−0.11	0.11
Myo-Tric-1F	0.01	**−0.37**	−0.08	0.22	0.16	0.01	0.01	−0.11	0.11
Myo-Tric-1S	**0.29**	**−0.26**	−0.25	0.09	0.20	−0.04	−0.13	0.10	−0.12
Myo-Tric-1D	**0.37**	0.10	−0.04	0.08	−0.08	−0.07	−0.08	0.06	−0.05
Myo-Tric-1R	0.18	0.11	−0.03	−0.22	−0.10	**0.32**	0.08	**0.27**	−0.01
Myo-Tric-1C	−0.20	−0.04	−0.15	0.02	−0.05	0.01	−0.07	0.09	0.08
Myo-Brarad-1F	0.21	**−0.28**	**−0.28**	0.08	0.09	−0.02	−0.14	−0.02	−0.18
Myo-Brarad-1S	0.10	**−0.44**	−0.18	0.05	0.14	−0.04	−0.03	−0.04	−0.05
Myo-Brarad-1D	−0.02	0.21	−0.09	−0.12	−0.10	**0.27**	−0.24	0.15	0.10
Myo-Brarad-1R	−0.03	−0.21	−0.15	−0.20	−0.15	0.12	0.02	0.09	−0.08
Myo-Brarad-1C	−0.05	0.03	0.20	0.05	−0.08	0.09	0.10	−0.12	0.01
Myo-Delt-1F	0.04	**−0.45**	−0.19	0.05	0.20	−0.03	−0.08	−0.23	−0.22
Myo-Delt-1S	0.13	**−0.45**	**−0.29**	0.15	0.17	−0.03	−0.03	−0.12	0.02
Myo-Delt-1D	−0.18	−0.18	−0.04	0.09	**0.32**	0.09	−0.10	−0.12	0.12
Myo-Delt-1R	0.14	−0.10	−0.01	−0.02	0.02	0.00	0.24	0.07	0.00
Myo-Delt-1C	**0.26**	0.09	0.11	0.09	0.12	−0.15	−0.25	−0.15	−0.12
Myo-Lat/Do-1F	0.14	**−0.49**	−0.14	0.20	0.07	0.01	−0.14	0.02	−0.09
Myo-Lat/Do-1S	0.24	**−0.37**	−0.25	0.01	0.08	−0.05	−0.15	−0.01	−0.15
Myo-Lat/Do-1D	0.04	−0.25	−0.23	0.12	0.16	**0.31**	0.25	−0.24	−0.01
Myo-Lat/Do-1R	−0.25	**−0.36**	0.13	**0.30**	0.07	0.02	−0.01	−0.20	**0.40**
Myo-Lat/Do-1C	0.21	−0.10	**−0.31**	0.12	0.23	0.07	−0.20	−0.22	0.07
Myo-Infra-1F	0.16	**−0.49**	**−0.46**	0.10	0.17	0.05	−0.06	0.04	−0.18
Myo-Infra-1S	0.21	**−0.40**	−0.17	0.01	0.04	−0.02	−0.06	−0.18	0.04
Myo-Infra-1D	−0.19	−0.08	0.00	−0.04	0.09	0.08	0.01	−0.03	0.13
Myo-Infra-1R	0.16	−0.10	−0.18	0.03	0.19	0.00	−0.25	−0.04	−0.18
Myo-Infra-1C	−0.14	−0.05	0.06	−0.07	0.11	0.04	0.03	0.00	0.02
Myo-Up.Tra-1F	0.07	**−0.37**	−0.01	0.16	0.13	0.11	0.00	0.00	0.12
Myo-Up.Tra-1S	0.07	**−0.55**	**−0.28**	0.22	**0.26**	−0.11	−0.11	−0.22	−0.03
Myo-Up.Tra-1D	**−0.28**	0.05	0.11	−0.21	−0.11	0.18	0.03	−0.16	−0.09
Myo-Up.Tra-1R	0.07	0.05	0.08	−0.09	**−0.28**	0.10	0.01	0.19	0.21
Myo-Up.Tra-1C	0.17	0.05	0.01	−0.05	0.03	0.20	0.15	0.03	0.15
Myo-FDS-1F	0.19	**−0.26**	−0.10	−0.04	0.16	−0.02	0.04	−0.03	−0.13
Myo-FDS-1S	0.08	**−0.44**	−0.17	0.05	0.11	0.05	−0.04	−0.09	−0.15
Myo-FDS-1D	0.24	−0.15	−0.10	−0.01	0.05	**0.36**	0.09	0.08	0.18
Myo-FDS-1R	0.17	0.03	−0.14	−0.10	−0.08	0.14	0.08	−0.01	−0.06
Myo-FDS-1C	0.00	0.00	−0.07	−0.06	**−0.28**	**0.29**	**0.28**	0.08	0.25
Myo-Pro-Te-1F	0.18	**−0.31**	−0.13	−0.11	0.10	0.04	−0.10	−0.10	−0.14
Myo-Pro-Te-1S	0.08	**−0.41**	−0.25	0.02	0.02	−0.25	−0.15	0.04	−0.16
Myo-Pro-Te-1D	0.07	0.07	−0.17	0.03	0.14	−0.11	0.02	−0.07	−0.07
Myo-Pro-Te-1R	0.18	0.06	0.12	0.08	0.03	0.03	−0.16	0.19	−0.03
Myo-Pro-Te-1C	−0.13	−0.05	0.02	−0.01	−0.16	0.14	−0.03	0.23	0.13
Myo-F.Ca-1F	0.19	**−0.33**	−0.23	0.10	0.04	−0.04	−0.16	−0.07	−0.04
Myo-F.Ca-1S	0.06	**−0.50**	**−0.33**	0.11	0.21	0.04	0.02	−0.09	−0.03
Myo-F.Ca-1D	**0.30**	0.01	**−0.27**	−0.25	−0.24	0.08	0.02	**0.28**	−0.09
Myo-F.Ca-1R	−0.05	0.16	0.11	**0.34**	−0.03	−0.06	−0.23	0.08	0.09
Myo-F.Ca-1C	−0.06	0.05	0.05	−0.06	−0.23	**0.33**	−0.09	0.01	0.11

Bold values indicate statistically significant correlations at *p* < 0.05. *ϱ* – Spearman rank correlation coefficient; Δ – mean change between the first and the last measurement; 1 – baseline value (first measurement). FMA-UE-Ttl – total score of upper limb motor function in the Fugl–Meyer Assessment Upper Extremity; Box n Blocks – Box and Block Test (gross manual dexterity test). EQ-5L – EuroQol 5 Dimensions 5 Levels questionnaire: EQ-5L-Por – mobility; EQ-5L-Sam – self-care; EQ-5L-Z/C – usual activities; EQ-5L-B/D – pain/discomfort; EQ-5L-N/P – anxiety/depression; EQ-5L-%Zd – self-rated health (EQ-VAS); EQ-5L-S.I – EQ-5D-5L summary index. Myo – biomechanical muscle parameters measured using the MyotonPRO device: F – frequency; S – stiffness; D – logarithmic decrement (elasticity); R – relaxation time; C – creep. Muscle abbreviations: Bic – biceps brachii; Tric – triceps brachii; Brarad – brachioradialis; Delt – deltoid; Lat/Do – latissimus dorsi; Infra – infraspinatus; Up.Tra – upper trapezius; FDS – flexor digitorum superficialis; Pro-Te – pronator teres; F.Ca – wrist flexors (flexor carpi muscles).

For changes in quality-of-life assessment, the highest number of correlations with baseline muscle tone values was found in the mobility domain (negative correlations with Myo-Brarad-1F, Myo-Delt-1S, Myo-Lat/Do-1C, Myo-Infra-1F, Myo-Up.Tra-1S, Myo-F.Ca-1S, Myo-F.Ca-1D) and in the pain/discomfort domain (positive correlations with Myo-Tric-1R, Myo-Brarad-1D, Myo-Lat/Do-1D, Myo-FDS-1D, Myo-FDS-1C, Myo-F.Ca-1C). All identified associations had at most a medium effect size. Higher baseline muscle tone values (for the respective parameters correlated with the indicated domains) were associated with smaller changes in mobility and greater changes in pain/discomfort. Changes in the self-care domain were positively correlated with baseline Myo-Lat/Do-1R and Myo-F.Ca-1R; in the usual activities domain with Myo-Delt-1D and Myo-Up.Tra-1S and negatively with Myo-Up.Tra-1R and Myo-FDS-1C; and in the anxiety/depression domain, positively with Myo-FDS-1C. Greater improvement in self-rated health was associated only with higher baseline Myo-Tric-1R and Myo-F.Ca-1D, while changes in the EQ-5L-S.I index were associated with Myo-Lat/Do-1R.

Correlation coefficients between baseline values of UL motor function and changes in muscle tone parameters and quality of life are presented in Table A3. Higher baseline values of FMA-UE-Ttl and Box n Blocks were associated only with smaller changes in Myo-F.Ca-C and greater changes in quality of life in the mobility domain. Associations between baseline FMA-UE-Ttl and Box n Blocks and non-significant changes in motor function were not considered.

## Discussion

The aim of the present study was to assess the correlations between UL motor function and changes in muscle tone as well as QoL in patients in the subacute phase after stroke. The obtained results indicate that improvement in UL motor function may occur largely independently of measurable changes in muscle tone parameters. However, the observed correlations were generally weak, and should therefore be interpreted with considerable caution. At the same time, improvements in motor performance appeared to show a clearer association with subjectively assessed QoL.

These findings suggest that recovery of UL function after stroke is a multifactorial process in which neuroplastic mechanisms and changes in motor control play a key role. Although increased muscle tone and spasticity are often considered significant factors limiting motor function after stroke, the present results suggest that their role in determining functional improvement may be more limited than traditionally assumed (62–64). Increasingly, spasticity is recognised as only one component of the complex spectrum of motor impairments following stroke, alongside factors such as muscle weakness, impaired motor coordination, and abnormal patterns of muscle activation (58).

An important aspect of the findings is the observed correlation between improvements in motor function and quality of life. This indicates that recovery of functional UL activity is important for daily functioning in patients after stroke. Improvements in manual dexterity and motor control may significantly influence independence in activities of daily living, social participation, and overall well-being (65, 66). UL functional limitations are among the most commonly reported problems by stroke patients and may persist for many months or even years following the neurological event.

The correlation between muscle tone abnormalities and functional recovery after stroke has been the subject of extensive discussion in the literature for many years. In the classical view, spasticity was regarded as one of the main factors limiting motor function in patients with upper motor neuron lesions. However, an increasing number of studies suggest that the correlation between muscle tone and functional abilities is considerably more complex (58).

In studies conducted by Sommerfeld et al., it was shown that although spasticity frequently develops after stroke, its severity does not always correlate directly with the level of UL functional impairment (37). Pandyan et al. had already emphasised that spasticity is a multifactorial phenomenon involving both neurological and biomechanical components (41). The complexity of spasticity is further confirmed by more recent publications (67–71). Considering these observations, muscle tone may influence movement quality, but it does not necessarily constitute the primary factor determining the ability to perform functional tasks.

In the present study, only isolated associations were observed between selected muscle tone parameters and changes in UL motor function. However, these correlations did not form a consistent pattern, suggesting that muscle tone is not the primary determinant of functional improvement in the analysed patient group. This observation is consistent with the concept that recovery of motor function after stroke results from adaptive processes occurring within the central nervous system.

The results of this study are also consistent with findings from stroke rehabilitation research. Ada et al. demonstrated that strengthening training can lead to improvements in motor function in stroke patients without a significant effect on spasticity levels (22). Similarly, Kwakkel et al. indicated that recovery of UL function is largely associated with the intensity and repetition of motor training (72).

In a systematic review on stroke rehabilitation, Veerbeek et al. also emphasised the importance of task-oriented therapy and intensive motor practice as key elements in improving UL function (73). These interventions are based on mechanisms of motor learning and experience-dependent brain plasticity.

From a neurophysiological perspective, these observations are consistent with the concept proposed by Krakauer et al., according to which recovery of motor function after stroke is largely a process of motor learning occurring under altered neurophysiological conditions (74).

Recovery of motor function after stroke is currently understood as a process resulting from the reorganisation of neural networks responsible for movement control (62, 63). Following focal brain injury, a range of adaptive processes occurs, including cortical reorganisation and changes in the functioning of neural networks (75).

One of the principal factors determining recovery of UL function is the integrity of the corticospinal tract. Neuroimaging studies have shown that preservation of at least part of the corticospinal projections is one of the strongest predictors of the recovery of voluntary movement after stroke (76). Reorganisation within cortical motor areas, such as the primary motor cortex, premotor cortex, and supplementary motor area, also plays a significant role (75, 76). During rehabilitation, functional reorganisation of these structures occurs, allowing preserved neuronal populations to assume control over movement.

At the same time, it should be emphasised that not all plastic processes are adaptive in nature. In some cases, so-called maladaptive plasticity develops, leading to abnormal movement patterns such as pathological muscle synergies or excessive co-activation of antagonist muscles (74, 77).

Sensory-motor integration also plays an important role in the recovery of motor function (78), as effective motor control depends on proper integration of sensory information with processes of movement planning and correction.

The findings of this study may have important implications for planning rehabilitation strategies in patients after stroke. These findings may help guide clinicians in prioritising rehabilitation strategies focused on functional training rather than solely targeting muscle tone reduction. In traditional therapeutic approaches, considerable emphasis has often been placed on reducing muscle tone as one of the primary treatment goals. However, the results of the present study suggest that focusing on reducing increased muscle tone may be insufficient to achieve meaningful functional improvements.

Rehabilitation strategies should place greater emphasis on improving motor control, active motor practice, and task-oriented training. Methods such as *constraint-induced movement therapy (CIMT),* intensive functional training, and robot-assisted rehabilitation may support neuroplastic processes and promote improvements in UL function (73, 79).

From the patient’s perspective, the correlation between improvements in motor function and quality of life is particularly important. Increased ability to perform daily activities may significantly enhance independence, social participation, and overall well-being in patients after stroke (65, 66).

Only a limited number of associations were observed between changes in muscle tone parameters and changes in quality of life, which may indicate that factors influencing the perception of quality of life after stroke are multidimensional and extend beyond the biomechanical properties of muscles alone.

An interesting observation was that some baseline muscle tone parameters were associated with the magnitude of subsequent changes in motor function and selected aspects of quality of life. This may suggest that the initial biomechanical properties of muscles may, to some extent, reflect the functional potential of the neuromuscular system and influence the course of the rehabilitation process.

In the correlation analysis, particular attention was drawn to the correlation between changes in the flexor digitorum superficialis muscle tone and changes in UL motor function. This muscle, together with the wrist flexors (flexor carpi radialis and flexor carpi ulnaris), plays a key role in functional grasp and object manipulation; therefore, increased tone in these muscles may contribute to limitations in performing precise manual tasks. The literature emphasises that spasticity affecting the finger flexors and wrist flexors is one of the most common patterns of motor impairment after stroke and is part of the characteristic flexor synergy pattern of the UL. These disturbances may limit the ability to open the hand and manipulate objects, thereby hindering functional use of the hand in activities of daily living (13, 37, 41, 80, 81). However, it should be emphasised that the observed correlations cannot be interpreted as causal correlations, but only as co-occurring changes between the analysed parameters.

In summary, the results suggest that improvement in UL function after stroke may occur independently of marked changes in muscle tone. However, given the generally weak correlations observed, these findings should be interpreted cautiously. Neuroplastic processes and mechanisms of motor learning may play an important role in functional recovery. Consequently, rehabilitation strategies should primarily focus on improving motor control and task-oriented functional training. Future research should further explore the correlations between changes in muscle tone, central nervous system reorganisation, and long-term functional outcomes of post-stroke rehabilitation.

## Limitations

This study has several limitations that should be considered when interpreting the results. First, the present study represents a secondary analysis of data derived from a randomized controlled trial, which limits the ability to draw causal inferences. Although the original study had an interventional design, the analysis of correlations between changes in muscle tone, motor function, and quality of life was not its primary objective. Therefore, the observed associations are correlational in nature and do not allow for definitive conclusions regarding causal correlations between the analysed variables.

Second, the study included patients in the subacute phase after stroke, which is characterised by particularly dynamic neuroplastic processes and substantial changes in motor function. The correlations between muscle tone and motor function may therefore differ at other stages of recovery, especially in the chronic phase, where secondary structural changes in muscles, tendons, and periarticular tissues may play a more prominent role.

Another limitation of the present study is the selection of outcome measures. Although widely used and validated tools such as the FMA-UE and the BBT were applied to assess motor function, other stroke-specific instruments, such as the Wolf Motor Function Test (WMFT) or the Action Research Arm Test (ARAT), might provide additional insight into UL functional performance. Similarly, QoL was assessed using the EQ-5D-5L questionnaire, which is a generic tool, while stroke-specific instruments such as the Stroke-Specific Quality of Life scale (SS-QoL) could allow for a more detailed evaluation of patient-reported outcomes. Future studies should consider incorporating more stroke-specific assessment tools to provide a more comprehensive evaluation of functional recovery and QoL.

In addition, the study did not include neuroimaging or neurophysiological assessments that could provide further insight into central nervous system reorganisation and corticospinal tract integrity. The inclusion of such methods in future research could contribute to a more comprehensive understanding of the mechanisms underlying the observed functional changes.

## Conclusion

The findings indicate a complex correlation between UL motor function, changes in muscle tone, and quality of life in patients in the subacute phase after stroke. Based on the conducted analysis, the following conclusions can be drawn:

1. Improvement in UL motor function in the subacute phase after stroke may occur independently of significant changes in muscle tone parameters, suggesting that muscle tone is not the primary determinant of functional recovery during this stage.
2. Improvement in motor function shows a stronger association with subjectively assessed quality of life, highlighting the importance of functional use of the UL in daily activities for patients after stroke.
3. The findings suggest that rehabilitation strategies focused on improving motor control and functional UL activity may be more effective in enhancing patient outcomes than interventions aimed solely at reducing muscle tone.

## Data overlap statement

Part of the dataset used in the present study (cohort *n* = 58, time points and assessment tools) overlaps with a previously published study evaluating the effectiveness of robotic and conventional UL therapy after stroke. However, the objectives, analytical approach, and reported outcomes of the present analysis differ from those of the earlier publication. In the current study, the intervention groups were not compared; instead, the entire cohort was analysed to examine the correlations between changes in muscle tone, UL motor function, and quality of life. The previous publication is cited as the source describing the intervention protocol.

## Data Availability

The original contributions presented in the study are included in the article/Supplementary material, further inquiries can be directed to the corresponding author.
