# Supplementary material for: Association of upper limb motor function with muscle tone changes and quality of life in the subacute phase after stroke: a prospective cohort study
Source: Front Neurol. 2026 May 25;17:1857094. doi: 10.3389/fneur.2026.1857094 (PMC13243092; doi:10.3389/fneur.2026.1857094)
Supplement: Supplementary file 1 [file Table_1.DOCX]

Supplementary Material

**Appendix 1.** Supplementary Materials

Table 2A. Changes in muscle tone parameters in post-stroke patients undergoing rehabilitation

|  | Pre-test | | Post-test | |  |  |  |  |
| --- | --- | --- | --- | --- | --- | --- | --- | --- |
|  | Mdn | IQR | Mdn | IQR | p | ES | ΔM±SD | 95%CI |
| Myo-Bic-F | 18.02 | 1.60 | 18.10 | 2.00 | 0.073 | 0.24 | 0.25 ± 1.17 | (-0.05; 0.56) |
| Myo-Bic-S | 304.55 | 28.40 | 302.20 | 26.50 | 0.620 | 0.07 | -0.56 ± 16.71 | (-4.96; 3.83) |
| Myo-Bic-D | 1.59 | 0.28 | 1.46 | 0.46 | **0.049** | 0.26 | -0.12 ± 0.41 | (-0.23; -0.01) |
| Myo-Bic-R | 18.90 | 6.40 | 22.10 | 4.70 | **0.003** | 0.39 | 2.26 ± 5.59 | (0.79; 3.73) |
| Myo-Bic-C | 1.54 | 0.70 | 1.80 | 0.53 | 0.067 | 0.24 | 0.13 ± 0.53 | (-0.01; 0.27) |
| Myo-Tric-F | 18.05 | 1.80 | 18.40 | 1.40 | 0.384 | 0.11 | -0.14 ± 1.2 | (-0.46; 0.17) |
| Myo-Tric-S | 303.05 | 28.40 | 303.15 | 29.50 | 0.102 | 0.22 | 4.03 ± 17.95 | (-0.69; 8.75) |
| Myo-Tric-D | 1.19 | 0.38 | 1.61 | 0.66 | **0.000** | 0.58 | 0.32 ± 0.45 | (0.2; 0.44) |
| Myo-Tric-R | 19.15 | 4.60 | 18.45 | 5.60 | 0.892 | 0.02 | 0.1 ± 5.06 | (-1.23; 1.43) |
| Myo-Tric-C | 1.47 | 0.61 | 1.55 | 0.61 | 0.627 | 0.06 | -0.02 ± 0.49 | (-0.15; 0.11) |
| Myo-Brarad-F | 18.20 | 2.10 | 18.50 | 2.10 | **0.027** | 0.29 | 0.31 ± 1.23 | (-0.01; 0.64) |
| Myo-Brarad-S | 304.55 | 25.60 | 303.25 | 30.80 | 0.634 | 0.06 | 1.12 ± 16.29 | (-3.16; 5.4) |
| Myo-Brarad-D | 1.54 | 0.21 | 1.59 | 0.47 | 0.272 | 0.14 | 0.07 ± 0.5 | (-0.07; 0.2) |
| Myo-Brarad-R | 19.20 | 4.20 | 19.70 | 5.50 | 0.194 | 0.17 | 0.84 ± 5.02 | (-0.48; 2.16) |
| Myo-Brarad-C | 1.54 | 0.62 | 1.48 | 0.55 | 0.389 | 0.11 | -0.07 ± 0.53 | (-0.2; 0.07) |
| Myo-Delt-F | 18.50 | 2.00 | 18.25 | 1.80 | 0.737 | 0.04 | -0.09 ± 1.25 | (-0.42; 0.24) |
| Myo-Delt-S | 305.55 | 25.90 | 305.40 | 23.10 | 0.693 | 0.05 | 0.91 ± 13.42 | (-2.62; 4.43) |
| Myo-Delt-D | 1.55 | 0.51 | 1.36 | 0.35 | 0.070 | 0.24 | -0.11 ± 0.47 | (-0.23; 0.01) |
| Myo-Delt-R | 18.60 | 4.60 | 18.75 | 5.90 | 0.858 | 0.02 | -0.14 ± 5.46 | (-1.57; 1.3) |
| Myo-Delt-C | 1.63 | 0.69 | 1.46 | 0.63 | 0.108 | 0.21 | -0.12 ± 0.54 | (-0.27; 0.02) |
| Myo-Lat/Do-F | 18.15 | 1.90 | 18.45 | 1.90 | 0.208 | 0.17 | 0.16 ± 1.2 | (-0.15; 0.48) |
| Myo-Lat/Do-S | 304.70 | 29.30 | 301.15 | 23.00 | 0.626 | 0.06 | -1.15 ± 14.74 | (-5.03; 2.73) |
| Myo-Lat/Do-D | 1.19 | 0.43 | 1.54 | 0.74 | **0.000** | 0.58 | 0.38 ± 0.53 | (0.23; 0.52) |
| Myo-Lat/Do-R | 19.00 | 6.40 | 20.70 | 5.10 | **0.035** | 0.28 | 1.54 ± 5.46 | (0.11; 2.98) |
| Myo-Lat/Do-C | 1.71 | 0.46 | 1.78 | 0.51 | **0.027** | 0.29 | 0.15 ± 0.46 | (0.03; 0.27) |
| Myo-Infra-F | 18.40 | 2.00 | 17.90 | 2.00 | **0.039** | 0.27 | -0.39 ± 1.31 | (-0.73; -0.04) |
| Myo-Infra-S | 303.45 | 27.60 | 299.90 | 29.70 | 0.572 | 0.07 | -1.06 ± 16.23 | (-5.33; 3.21) |
| Myo-Infra-D | 1.32 | 0.71 | 1.57 | 0.50 | **0.000** | 0.48 | 0.31 ± 0.58 | (0.16; 0.46) |
| Myo-Infra-R | 19.05 | 4.50 | 19.85 | 4.00 | 0.363 | 0.12 | 0.72 ± 5.04 | (-0.61; 2.04) |
| Myo-Infra-C | 1.70 | 0.68 | 1.53 | 0.63 | 0.053 | 0.25 | -0.14 ± 0.53 | (-0.28; 0) |
| Myo-Up.Tra-F | 18.35 | 1.80 | 18.30 | 2.10 | 0.052 | 0.26 | -0.21 ± 1.16 | (-0.51; 0.09) |
| Myo-Up.Tra-S | 307.65 | 25.90 | 305.55 | 27.60 | 0.772 | 0.04 | -0.08 ± 15.72 | (-4.22; 4.05) |
| Myo-Up.Tra-D | 1.45 | 1.03 | 1.46 | 1.13 | 0.448 | 0.10 | 0.07 ± 0.82 | (-0.15; 0.29) |
| Myo-Up.Tra-R | 19.35 | 4.40 | 20.35 | 4.50 | 0.206 | 0.17 | 0.77 ± 5.83 | (-0.76; 2.3) |
| Myo-Up.Tra-C | 1.70 | 0.59 | 1.79 | 0.53 | 0.079 | 0.23 | 0.11 ± 0.49 | (-0.02; 0.24) |
| Myo-FDS-F | 18.10 | 2.60 | 18.40 | 1.60 | 0.760 | 0.04 | -0.05 ± 1.31 | (-0.39; 0.3) |
| Myo-FDS-S | 304.05 | 27.50 | 302.45 | 27.10 | 0.518 | 0.08 | 1.25 ± 16.6 | (-3.12; 5.62) |
| Myo-FDS-D | 1.20 | 0.39 | 1.50 | 0.46 | **0.001** | 0.44 | 0.22 ± 0.46 | (0.1; 0.34) |
| Myo-FDS-R | 18.05 | 3.60 | 20.35 | 6.10 | **0.000** | 0.47 | 2.61 ± 5.45 | (1.17; 4.04) |
| Myo-FDS-C | 1.48 | 0.63 | 1.69 | 0.68 | 0.083 | 0.23 | 0.12 ± 0.54 | (-0.02; 0.26) |
| Myo-Pro-Te-F | 18.40 | 1.70 | 18.20 | 1.80 | 0.923 | 0.01 | -0.02 ± 1.3 | (-0.36; 0.32) |
| Myo-Pro-Te-S | 306.80 | 21.00 | 303.90 | 29.50 | 0.359 | 0.12 | -2.23 ± 16.85 | (-6.66; 2.2) |
| Myo-Pro-Te-D | 1.38 | 0.67 | 1.42 | 0.78 | 0.862 | 0.02 | -0.03 ± 0.72 | (-0.22; 0.16) |
| Myo-Pro-Te-R | 19.15 | 3.60 | 19.85 | 4.90 | 0.289 | 0.14 | 0.6 ± 5.79 | (-0.93; 2.12) |
| Myo-Pro-Te-C | 1.47 | 0.60 | 1.63 | 0.56 | 0.460 | 0.10 | 0.05 ± 0.51 | (-0.09; 0.18) |
| Myo-F.Ca-F | 18.15 | 1.50 | 17.95 | 2.10 | 0.714 | 0.05 | 0.08 ± 1.14 | (-0.22; 0.38) |
| Myo-F.Ca-S | 305.30 | 33.60 | 301.35 | 24.50 | 0.957 | 0.01 | -0.16 ± 17.01 | (-4.63; 4.31) |
| Myo-F.Ca-D | 1.55 | 0.34 | 1.29 | 0.34 | **0.000** | 0.52 | -0.26 ± 0.43 | (-0.37; -0.15) |
| Myo-F.Ca-R | 17.95 | 5.10 | 19.25 | 4.50 | 0.263 | 0.15 | 0.88 ± 5.66 | (-0.61; 2.37) |
| Myo-F.Ca-C | 1.36 | 0.57 | 1.73 | 0.55 | **0.001** | 0.42 | 0.23 ± 0.5 | (0.1; 0.36) |

Bold values indicate statistically significant differences at p < 0.05. Mdn – median; IQR – interquartile range; p – p-value (result of the Wilcoxon signed-rank test); ES – effect size; ΔM±SD – mean change and standard deviation of change; 95% CI – confidence interval. Myo – biomechanical muscle parameters measured using the MyotonPRO device: F – frequency; S – stiffness; D – logarithmic decrement (elasticity); R – relaxation time; C – creep. Muscle abbreviations: Bic – biceps brachii; Tric – triceps brachii; Brarad – brachioradialis; Delt – deltoid; Lat/Do – latissimus dorsi; Infra – infraspinatus; Up.Tra – upper trapezius; FDS – flexor digitorum superficialis; Pro-Te – pronator teres; F.Ca – wrist flexors (flexor carpi muscles).

Table 3A. Correlations (Spearman’s ϱ) between baseline upper limb motor function values and changes in muscle tone parameters and quality of life in post-stroke patients undergoing rehabilitation

Bold values indicate statistically significant correlations at p < 0.05. ρ – Spearman’s rank correlation coefficient; Δ – mean change between the first and the last measurement; –1 indicates baseline value; –d indicates change between the first and the last measurement. FMA-UE-Ttl – total score of upper limb motor function in the Fugl–Meyer Assessment Upper Extremity; Box n Blocks – Box and Block Test (gross manual dexterity test). EQ-5L – EuroQol 5 Dimensions 5 Levels questionnaire: EQ-5L-Por – mobility; EQ-5L-Sam – self-care; EQ-5L-Z/C – usual activities; EQ-5L-B/D – pain/discomfort; EQ-5L-N/P – anxiety/depression; EQ-5L-%Zd – self-rated health (EQ-VAS); EQ-5L-S.I – EQ-5D-5L summary index. Myo – biomechanical muscle parameters measured using the MyotonPRO device: F – frequency; S – stiffness; D – logarithmic decrement (elasticity); R – relaxation time; C – creep. Muscle abbreviations: Bic – biceps brachii; Tric – triceps brachii; Brarad – brachioradialis; Lat/Do – latissimus dorsi; Infra – infraspinatus; FDS – flexor digitorum superficialis; F.Ca – wrist flexors (flexor carpi muscles).

|  | FMA-UE-Ttl-1 | Box n Blocks-1 |
| --- | --- | --- |
| ΔMyo-Bic-D | -0.09 | -0.04 |
| ΔMyo-Bic-R | 0.07 | 0.05 |
| ΔMyo-Tric-D | 0.02 | 0.01 |
| ΔMyo-Brarad-F | -0.09 | -0.11 |
| ΔMyo-Lat/Do-D | 0.02 | 0.04 |
| ΔMyo-Lat/Do-R | 0.04 | 0.11 |
| ΔMyo-Lat/Do-C | -0.22 | -0.22 |
| ΔMyo-Infra-F | -0.17 | -0.21 |
| ΔMyo-Infra-D | -0.01 | -0.11 |
| ΔMyo-FDS-D | 0.13 | 0.03 |
| ΔMyo-FDS-R | 0.10 | 0.14 |
| ΔMyo-F.Ca-D | 0.20 | 0.09 |
| ΔMyo-F.Ca-C | **-0.29** | **-0.32** |
| EQ-5L-Por-d | **0.39** | **0.29** |
| EQ-5L-Sam d | 0.05 | -0.03 |
| EQ-5L-Z/C-d | -0.11 | -0.13 |
| EQ-5L-B/D-d | 0.02 | 0.11 |
| EQ-5L-N/P-d | 0.07 | 0.07 |
| EQ-5L-%Zd-d | -0.12 | -0.09 |
| EQ-5L-S.I-d | 0.22 | 0.17 |
